# Comparison of ROTEM parameters from venous and intraosseous blood

**DOI:** 10.1038/s41598-019-40412-0

**Published:** 2019-03-06

**Authors:** Marion Wiegele, Thomas Hamp, Johannes Gratz, Eleonore Pablik, Eva Schaden

**Affiliations:** 1 Department of Anaesthesia, Critical Care and Pain Medicine, Division of General Anaesthesia and Intensive Care Medicine, Medical University of Vienna, Waehringer Guertel 18-20, 1090 Vienna, Austria; 2 Center for Medical Statistics, Informatics, and Intelligent Systems, Section for Medical Statistics, Spitalgasse 23, 1090 Vienna, Austria

## Abstract

Rotational thromboelastometry is recommended to guide haemostatic therapy in trauma-related coagulopathy. In the case of unsuccessful venepuncture, intraosseous access allows immediate administration of drugs and volume replacement. Feasibility of rotational thromboelastometry from intraosseous blood has not yet been investigated in humans. We performed rotational thromboelastometry and standard coagulation assays from intraosseous and intravenous blood samples in 19 volunteers and 4 patients undergoing general anaesthesia. Intraosseous access was performed either at the tibial bone or the proximal humerus. We observed visible clotting in the majority of the intraosseous samples. Only 13% of the probes allowed realization of rotational thromboelastometry. ROTEM parameters are reported as follows: shorter median clotting time (CT) in EXTEM, INTEM, and APTEM (53 vs. 68 s; 140 vs. 154 s; 54 vs. 62.5 s) and smaller median maximal clot firmness (MCF) in EXTEM and APTEM (56 vs. 63 mm; 55 vs. 62 mm) in intraosseous samples. We found no relevant differences in median MCF values in FIBTEM and INTEM (12 vs. 13 mm; 60 vs. 59 mm). Given the difficulties we faced during IO blood sampling in a study setting, we advise against ROTEM measurements out of IO blood for guidance of procoagulant therapy in emergency situations.

## Introduction

Establishing intravenous (IV) access can be challenging in severely injured patients. In the case of difficult venepuncture, current guidelines recommend the intraosseous (IO) route for drug administration and volume replacement^1^.

Despite declining incidence of trauma-induced coagulopathy, severe bleeding still affects mortality and requires immediate intervention^2,3^. Viscoelastic tests such as rotational thromboelastometry (ROTEM) allow a goal-directed, individualized approach to haemostatic therapy in trauma patients^3–5^.

Results of current trials confirm the feasibility and effectiveness of IO administration of blood products, tranexamic acid, and fibrinogen^6–8^. A case report showed the successful IO administration of prothrombin complex concentrate^9^. Moreover, the reversal of dabigatran using IO idarucizumab in a porcine trauma model has been reported^10^. Hence, it seems appropriate to investigate the reliability of ROTEM parameters determined in IO blood to allow a target-oriented administration of procoagulant drugs in patients who have only IO access.

A recent study by Strandberg *et al*. investigated the feasibility of measuring coagulation parameters including ROTEM from IO blood in a swine model^11^. However, to the best of our knowledge, data from human studies are not available so far. Therefore, the aim of our study was to compare ROTEM parameters determined in blood from IO and venous access in healthy individuals and patients without coagulopathy.

## Materials and Methods

### Study setting

This prospective observational study was performed at the General Hospital of Vienna, Austria, from June 2015 to November 2017 in accordance with the Declaration of Helsinki. Ethics Committee approval (Ethics Committee of the Medical University of Vienna, Austria, 1699/2014) was obtained, and the study was registered at the German Clinical Trials Register (DRKS00007902) before participants were enrolled. Written informed consent was obtained from all participants prior to enrolment.

The study was supported by a research grant from the Austrian Association for Anaesthesia, Resuscitation and Intensive Care Medicine (ÖGARI). Teleflex Inc. provided the equipment for IO accesses.

The funders had no role in the design of the study; the collection, analysis, or interpretation of data; or writing of the manuscript.

### Study population

In the first part of our study, we enrolled healthy volunteers. In the second part, we included patients undergoing minor surgery under general anaesthesia.

Prior to inclusion, we obtained the participants’ medical and bleeding history from a standardized questionnaire to rule out potential bleeding disorders or the ingestion of substances that could affect coagulation tests^12^. Volunteers and patients with suspected or known coagulation disorders based on the standardized questionnaire as well as pregnant and breast-feeding patients were excluded.

### Study procedures

#### IO, IV access, and blood sampling

Prior to establishing IO access under sterile conditions, venous access was established in all patients using an 18GA peripheral venous cannula (BD Vialon, Becton Dickinson Infusion Therapy, AB, SE-251 Helsingborg, Sweden).

At the beginning of the study period, we established IO access in the proximal tibia after subcutaneous administration of 2% lidocaine-hydrochloride using the appropriate 25-millimetre (mm) needles of the Arrow® EZ-IO® Intraosseous Infusion System Device (Vidacare Corporation, San Antonio, Texas, USA). As recommended by the manufacturer, the correct position of the IO needle was verified by aspiration of blood and subsequent flush with sodium chloride 0.9% immediately after insertion.

Despite correct position of the IO needle, we experienced difficulties in obtaining adequate blood samples, as blood clotted visibly in the vast majority of patients.

To overcome this problem, we modified the sampling technique repeatedly.

First, we changed the blood-sampling system. Blood flow through the IO system was very slow when we used our routine blood-sampling system (Vacuette™, Greiner, Kremsmünster, Austria). We decided to switch to S-Monovette^®^ (Sarstedt, Nümbrecht, Germany) tubes, which allow a modification of aspiration flow as there is no fixed vacuum within the tube. As low blood flow and early clotting persisted, we finally switched to aspirating IO blood using plastic syringes, which allowed higher suction forces, and transferred the blood immediately into the tubes for coagulation testing. However, it was not possible to increase blood flow, and the IO blood continued to clot visibly.

Second, we changed the IO access site. Pasley *et al*. reported higher flow rates at IO infusion when the proximal humerus was used instead of the proximal tibia^13^. Thus, after failing to get adequate blood samples from the first seven patients, we switched the IO access insertion site to the proximal humerus using the required 45-mm needles. However, many volunteers reported severe pain during aspiration, which limited the amount of suction force we could apply. Therefore, we decided to flush the IO system with 2% lidocaine-hydrochloride after insertion as recommended, which did not prove helpful either^14–16^.

Consequently, in a final step, the IO access was performed under general anaesthesia in four patients to rule out the influence of pain on flow rates and thus clotting during blood sampling.

Two millilitres of blood were wasted before taking the samples for analysis^17^. Samples for conventional coagulation assays and ROTEM were drawn in tubes containing trisodium citrate 3.8%. Samples for the platelet count were drawn into ethylenediaminetetraacetic acid (EDTA) tubes.

We attempted to obtain IO and venous blood samples from each participant. All samples that were not clotted visibly were analysed immediately.

#### ROTEM

ROTEM tests (TEM Innovations, Munich, Germany) were performed in citrated whole blood according to the manufacturer’s instructions. A total of 300 microlitre (μL) of citrated whole blood was mixed with 20 μL of EXTEM, INTEM, FIBTEM, or APTEM reagent, and coagulation was started with 20 μL CaCl_2_ 0.2 millimol per liter (mmol l^−1^). All samples were prepared with the automated pipette integrated in the ROTEM, which allows accurate dosage of volumes between 20 and 300 μL. ROTEM quality control measurements were performed once weekly as recommended by the manufacturer. In the EXTEM, INTEM, and APTEM test, the main endpoints were clotting time (CT), maximal clot firmness (MCF), and clot formation time (CFT). Furthermore, the alpha angle (α), maximum lysis (ML), and clot firmness after 10 minutes (A10) were analysed. In the FIBTEM test, MCF and A10 were determined.

#### Conventional coagulation assays (CCA) and platelet count

The following coagulation tests were assessed from citrated plasma: prothrombin time (PT; Owren, given as percentage of normal, reference value 70–125%), activated partial thromboplastin time (aPTT; given in seconds, reference value 27–41 s), and the fibrinogen level [Clauss method, given in milligram per decilitre (mg dl^−1^), reference value 200–400 mg dL^−1^]. All tests were performed using the STA-R Evolution^®^ coagulometer (Diagnostica Stago S.A.S., Asnières sur Seine, France). The platelet count [given in Giga per litre (G L^−1^), reference value 150–350 G L^−1^] was assessed from an EDTA tube with a Sysmex XE- 2100 cell counter (Sysmex, Kobe, Japan).

#### Patient data

We documented age, sex, body mass index, medical history, and results of bleeding history prior to inclusion into the study. All participants underwent periprocedural hemodynamic monitoring (heart rate, non-invasive blood pressure, saturation of peripheral oxygen).

Periprocedural pain was assessed using the Numeric Rating Scale (NRS; reference range 0–10 points) at the following time points: baseline (t0), placement of IV access (t1), placement of IO access (t2), aspiration from/injection into the IO access (t3), and the end of the procedure (t4).

### Ethics approval and consent to participate

The study has been approved by the appropriate institutional ethics committee [Ethics Committee of the Medical University of Vienna, Austria, EK 1699/2014] and registered at the German Clinical Trials Register (DRKS00007902). Informed consent was obtained from all individual participants included in the study. All procedures have been performed in accordance with the ethical standards as laid down in the 1964 Declaration of Helsinki and its later amendments or comparable ethical standards.

### Statistical methods

Descriptive summaries for platelet count and CCA results in the IO and IV blood samples as well as for their difference are given as median (first quartile; third quartile). A significant difference between the two samples was evaluated with the Wilcoxon signed rank test, and the correlation between the two measurements was calculated with Spearman’s correlation coefficient. For all tests, values of *p* < 0.05 were considered to be significant without correction for multiple testing and therefore must be interpreted as hypothesis-generating only. The median (first quartile, third quartile) was also reported for the IV ROTEM parameters. Because of the small number of IO blood samples eligible for all four ROTEM tests, we presented median (min, max) values for these measurements and their differences to the IV values. Note that these three values directly correspond to the three individual measurements observed.

Data were analysed with R version 3.4.3.

## Results

### Patient characteristics

We enrolled a total of 23 participants: 19 in the first (local anaesthesia) and 4 in the second (general anaesthesia) part of our study.

Participants’ characteristics and morphometric data are shown in Table 1.

**Table 1:** Descriptive statistics and results of pain scores (Numeric Rating Scale [NRS]) for volunteers and patients (f, female; m, male; age [years]; BMI, body mass index; SD, standard deviation).

	volunteers	patients
gender	f:m	f:m
	9:10	1:3
	Mean (SD)	Mean (SD)
age	25 (8)	39 (20)
BMI	22 (5)	22 (3)
NRS t0	0 (0)	—
NRS t1	1 (1)	—
NRS t2	3 (2)	—
NRS t3	6 (3)	—
NRS t4	0 (1)	—

Volunteers reported a mean NRS of 6 points (min 1 point, max 9 points) during IO aspiration or injection (Table 1).

### Blood sampling

We obtained usable blood samples from IO access in 39.1% (9/23) of participants. In the remaining participants, the blood clotted visibly during the sampling procedure. The success rate for obtaining an IO sample did not differ between the access sites or between participants receiving local or general anaesthesia for the procedure.

Blood from IV access was usable in all participants.

#### ROTEM

Plausible results with ROTEM from IO samples could be obtained in 33% (3/9) of the cases where IO sampling was initially successful. This translates to an overall success rate of 13% (3/23) for obtaining a plausible ROTEM result from IO sampling.

ROTEM analysis of IV samples was performed in 17 of the participants, and plausible results were obtained in all cases.

A direct comparison of ROTEM analysis of IO to IV samples was possible in three cases. The results are presented in Table 2.

**Table 2:** Results of IO and IV ROTEM parameters in EXTEM, FIBTEM, INTEM, and APTEM tests (CT, clotting time[s]; CFT, clot formation time[s]; MCF, maximum clot firmness [mm]; alpha, alpha-angle; A10, amplitude time point of 10 minutes after CT [mm]; ML, maximum lysis [%]; IO, intraosseous; IV, intravenous; min, minimum; max, maximum; 1.Q, first quartile; 3.Q, third quartile).

	IV(n = 17)		IO (n = 3)		diff IO-IV (n = 3)	
	median	(1.Q;3.Q)	median	(min;max)	median	(min;max)
EXTEM CT	68	(60;76)	53	(52;64)	4	(4;14)
EXTEM CFT	86	(76;106)	117	(75;148)	9	(0;70)
EXTEM MCF	63	(58;66)	56	(54;66)	−2	(−17;0)
EXTEM alpha	72	(70;76)	70	(67;77)	−1	(−4;0)
EXTEM A10	55	(49;58)	49	(46;59)	−1	(−16;2)
EXTEM ML	6	(2;9)	9	(7;12)	3	(0;12)
FIBTEM A10	12.5	(10;15.75)	12	(9;14)	−5	(−6;0)
FIBTEM MCF	13	(10;17.75)	12	(10;14)	−7	(−7;0)
APTEM CT	62.5	(53.5;65.5)	54	(51;59)	6	(−3;18)
APTEM CFT	96.5	(79.75;109.25)	115	(71;131)	−2	(−13;26)
APTEM MCF	62	(58;64)	55	(54;64)	−5	(−14;1)
APTEM alpha	72	(69;74.5)	68	(67;76)	−1	(−1;0)
APTEM A10	54	(49.75;56)	47	(45;57)	−3	(−11;3)
APTEM ML	6	(0.5;10)	7	(0;10)	1	(0;3)
INTEM CT	154	(144;171)	140	(117;166)	−30	(−77;−4)
INTEM CFT	80	(62;92)	72	(60;132)	−9	(−20;40)
INTEM MCF	59	(58;65)	60	(53;64)	1	(−16;2)
INTEM alpha	74	(72;77)	76	(68;78)	2	(−3;4)
INTEM A10	56	(51;58)	55	(45;59)	3	(−13;3)
INTEM ML	6	(2.5;10.25)	6	(3;8)	2	(−7;7)

#### Conventional coagulation assays

We determined PT, aPTT, international normalized ratio (INR), and fibrinogen in 39.1% (9/23) of IO and 86.9% (20/23) of IV blood samples. There was a median difference of −4.5% for PT, −3.5 s for aPTT, 0 for INR, and −74.5 mg/dL for fibrinogen. A moderate to good correlation between the IO and IV measurements was found for PT, aPTT, and INR (ρ = 0.84; ρ = 0.75; ρ = 0.62). The correlation between the IO and IV fibrinogen values was very poor (ρ = −0.14; Table 3).

**Table 3:** Results of conventional coagulation assays and platelet count in IO and IV blood samples (platelet count [G L^−1^]; TT, thromboplastin time[s]; PT, prothrombin time [%]; aPTT, activated partial thromboplastin time[s]; INR, international normalized ratio; fibrinogen [mg dL^−1^]; IO, intraosseous; IV, intravenous).

	n	Median	i.v.	n	Median	i.o.	n	Median	Diff i.o. – i.v.	Wilcox.	Spearman	
			(1.Q;3.Q)			(1.Q;3.Q)			(1.Q;3.Q)	*p* value	Rho	*p* value
Platelet count	20	236.5	(201.5;265.25)	9	102	(24;161)	9	–147	(−176; −84)	0.0039	0.283	0.463
PT	19	79	(77;94)	9	75	(57;120)	8	−4.5	(−12.5; 0.25)	0.1824	0.838	0.0093
aPTT	19	36.3	(32.9;38.3)	8	33.6	(27;86.2)	7	−3.5	(−5.15; 0.05)	0.4688	0.75	0.0663
Fibrinogen	19	258	(222.5;291)	7	187	(1;288)	6	−74.5	(−167; −11.25)	0.1563	−0.145	0.7841

#### Platelet count

A comparison of platelet count in IO and IV samples was possible in 9 cases (39.1%). We found a significantly lower platelet count in IO samples and a poor correlation (ρ = 0.28) between the IO and IV results (Table 3).

## Discussion

To the best of our knowledge, this is the first study presenting results of coagulation studies out of human IO blood. In our study, ROTEM obtained from IO blood samples was feasible in only 3 out of 23 participants. Early clotting within the tubes averted further analysis or led to implausible results in all other probes. Therefore, results of ROTEM parameters out of IO and IV samples could not be compared in a statistically reliable manner as planned. However, the unintended but major finding of our study is that ROTEM measurements out of IO blood are not reliable and should not be used for guidance of procoagulant therapy in the emergency setting.

Several studies investigated standard laboratory parameters from human IO blood samples^17–20^. The authors found significant correlations for red blood cell counts, haemoglobin levels, and the haematocrit determined in IO and IV blood. Furthermore, a comparatively lower platelet count in blood samples obtained from IO access has been described, which is in line with the findings of our study. We analysed platelet counts in 9 out of 23 participants, and results showed a significantly lower platelet count in IO blood samples (Table 3). Serum chemistry parameters (sodium, glucose, blood urea nitrogen, creatinine, chloride, total protein, and albumin concentrations) have also been comparable between IO and IV blood^17–19^. Conflicting results have been shown regarding the calcium level only. Hurren and colleagues reported similar calcium levels in IO and IV blood samples; other trials found no correlation^17–19^.

Notably, to date, no human studies evaluating haemostatic parameters from IO blood have been published. In 2016, Strandberg and colleagues compared CCAs und parameters of thromboelastography (TEG) from IO and IV blood samples in a porcine, haemodiluted model^11^. They reported similar problems with early clotting: fewer than half of the CCA samples allowed laboratory analysis. In contrast to our study, TEG could be performed in the majority of the probes. The authors found shorter R times (representing faster activation) and lower MA amplitudes (indicating consumption) in the IO samples and assumed a “clinical hypercoagulability” within the IO samples They concluded that analysis of coagulation in human IO aspirate might perform differently from that in anaesthetized pigs and recommended human trials.

Although parameters of TEG and ROTEM are not interchangeable regarding the reference values, the reaction time (R) in TEG and CT in ROTEM are similar, as both provide information about initial thrombin generation and fibrin formation. The maximum amplitude in TEG and the MCF in ROTEM depend on the fibrinogen level and platelet count. In terms of the reagent used, INTEM is the ROTEM test modification that is best comparable with TEG. Results of the animal study showed a shorter R in TEG in IO samples, which is in line with the results of the INTEM test in our study (Table 2). In contrast to Strandberg *et al*., we found an almost equal median INTEM MCF in IO and IV blood samples^11^. However, MCF of EXTEM and APTEM revealed the expected smaller IO median amplitude, which corresponds to lower IO platelet counts and fibrinogen levels found in all IO samples and could be interpreted as premature activation of the coagulation process.

PT revealed a good reproducibility between IO and IV samples in human and animal studies, whereas fibrinogen levels were not comparable in both. Conflicting results exist for aPTT: while we found a good correlation, clinically relevant differences were found in the porcine model.

**Figure 1:**
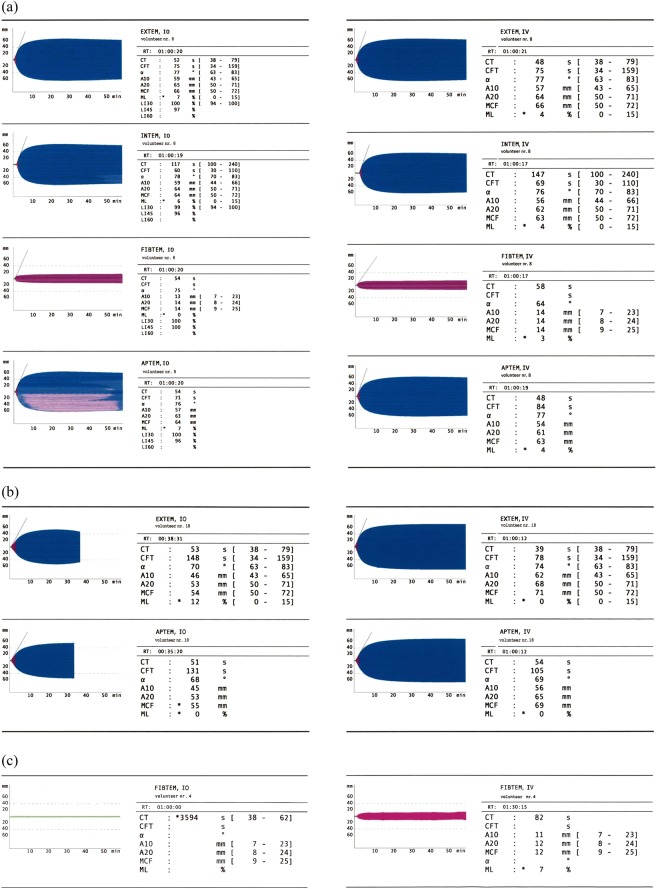
IO and IV ROTEM test results in healthy volunteers: whereas one probe reveals reliable IO ROTEM test results when compared to IV measurements (**a**), another – putative unclotted probe – might be interpreted as “late hyperfibrinolysis” although the IV sample shows normal test results (**b**). Due to early clotting of the sample, the trace of the third IO FIBTEM test resembles afibrinogenaemia (**c**).

Early clotting within the tubes remained the major problem during IO blood sampling. As described above, we tried to systematically exclude potential procedural and operating errors. However, this «side effect» is probably the major finding of our study, as ROTEM results from clotted blood could be misinterpreted as a coagulation breakdown in the patient and lead to unnecessary and potentially harmful procoagulant therapies. Figure 1 depicts the broad variation of test results: whereas one probe reveals reliable IO ROTEM test results when compared to IV measurements, another – putative unclotted probe – might be interpreted as “late hyperfibrinolysis” although the IV sample shows normal test results. Due to early clotting of the sample, the trace of the third IO FIBTEM test resembles afibrinogenaemia. For experienced users, the difference should be clear at first sight, but ROTEM is often used in emergency situations, which makes errors more likely.

Our study has some relevant limitations. First, the sample size is small. Despite multiple periprocedural adjustments, we were unable to obtain a higher number of usable blood samples and decided to terminate the study prematurely. Second, we did not perform CCAs from IV blood in all participants, because clotting of the IO sample would not have allowed comparison of parameters. Third, we did not measure calcium levels in IO samples. A very high calcium level in the IO aspirate might have contributed to the observed hypercoagulability.

## Conclusion

The present study is the first to show results of ROTEM and conventional coagulation assays determined in IO blood from volunteers and patients. We observed early clotting in more than 60% of collected blood samples leading to unreliable results.

Given the difficulties we faced during IO blood sampling in a study setting, we advise against ROTEM measurements out of IO blood for guidance of procoagulant therapy in emergency situations.

## Data Availability

The datasets supporting the conclusions of this article are included within the article and its additional files. Further data are available from the corresponding author on reasonable request.
